# Interleukin-6 downregulation with mesenchymal stem cell differentiation results in loss of immunoprivilege

**DOI:** 10.1111/jcmm.12092

**Published:** 2013-06-26

**Authors:** Peng Li, Shu-Hong Li, Jun Wu, Wang-Fu Zang, Sanjiv Dhingra, Lu Sun, Richard D Weisel, Ren-Ke Li

**Affiliations:** a Department of Cardiac Surgery Rui Jin Hospital, Shanghai Jiao Tong University School of MedicineShanghai, China; b Division of Cardiovascular Surgery and Toronto General Research Institute University Health Network and Department of Surgery Division of Cardiac Surgery, University of TorontoToronto, ON, Canada

**Keywords:** interleukin-6, immunoprivilege, mesenchymal stem cell, cell differentiation, immune rejection

## Abstract

Allogeneic mesenchymal stem cell (MSC) transplantation improves cardiac function, but cellular differentiation results in loss of immunoprivilege and rejection. To explore the mechanism involved in this immune rejection, we investigated the influence of interleukin-6 (IL-6), a factor secreted by MSCs, on immune privilege after myogenic, endothelial and smooth muscle cell differentiation induced by 5-azacytidine, VEGF, and transforming growth factor-β (TGF-β), respectively. Both RT-PCR and ELISA showed that myogenic differentiation of MSCs was associated with significant downregulation of IL-6 expression (*P* < 0.01), which was also observed following endothelial (*P* < 0.01) and smooth muscle cell differentiation (*P* < 0.05), indicating that IL-6 downregulation was dependent on differentiation but not cell phenotype. Flow cytometry demonstrated that IL-6 downregulation as a result of myogenic differentiation was associated with increased leucocyte-mediated cell death in an allogeneic leucocyte co-culture study (*P* < 0.01). The allogeneic reactivity associated with IL-6 downregulation was also observed following MSC differentiation to endothelial and smooth muscle cells (*P* < 0.01), demonstrating that leucocyte-mediated cytotoxicity was also dependent on differentiation but not cell phenotype. Restoration of IL-6 partially rescued the differentiated cells from leucocyte-mediated cell death. These findings suggest that rejection of allogeneic MSCs after implantation may be because of a reduction in cellular IL-6 levels, and restoration of IL-6 may be a new target to retain MSC immunoprivilege.

## Introduction

Cardiomyocytes have limited regenerative capability following myocardial ischemic injury, and the loss of these cells can result in a cascade of events that lead to heart failure [1]. Stem cell therapy has been studied extensively, but the ideal cell type has not been determined. Mesenchymal stem cells (MSCs) are attractive candidates for cell therapy because they are multipotent, readily available and can be expanded in culture. However, perhaps their most intriguing feature is their immunoprivilege, which stems in part from their unique expression of cell surface major histocompatibility complex (MHC) antigens. Mesenchymal stem cells express low levels of MHC-Ia, which is immunogenic, high levels of MHC-Ib, which is immunosuppressive, and no MHC-II, which is an alloantigen [2–4]. In addition, MSCs have extensive regenerative potential because of their release of cytokines and their ability to differentiate into myogenic, smooth muscle and endothelial cells, which participate in tissue restoration [5–7]. As the quantity and quality of MSCs are diminished with age [8, 9], the use of allogeneic donor MSCs for cell therapy and tissue regeneration is being extensively investigated.

Allogeneic MSCs have been used successfully for myocardial regeneration in animals [10], and the clinical use of these cells to restore cardiac function after a myocardial infarction (MI) has been reported [11, 12]. We previously demonstrated that allogeneic MSC transplantation improved cardiac function early after an MI, but later, following myogenic differentiation, the cells were no longer immunoprivileged and were rejected, resulting in the deterioration of ventricular function [2]. We found that MSC differentiation was accompanied by changes in the expression of the cells’ MHC antigens: increased MHC-Ia and MHC-II, and decreased MHC-Ib [2].

In addition to the contribution of cell surface antigens, the immunoprivilege of MSCs is also maintained by their production of a number of soluble immunosuppressive factors, including indoleamine 2,3-dioxygenase (IDO), interleukin-6 (IL-6) and prostaglandin E2 (PGE2) [13–16]. IL-6 is secreted by MSCs [13, 15] and is believed to be a pro-inflammatory cytokine. Recently, however, IL-6 has been reported to be involved in the suppression of T cell proliferation and local inflammation [17, 18], and MSC-derived IL-6 was reported to contribute to immunoregulatory activity [19, 20]. Based on these reports, IL-6 appears to be involved in a series of immunosuppressive actions and perhaps could be involved in the rejection of allogeneic MSCs following implantation.

Accordingly, we investigated whether the secretion of IL-6 changed following the differentiation of MSCs. We induced myogenic, endothelial and smooth muscle cell differentiation of MSCs and performed allogeneic leucocyte co-culture experiments to evaluate the relationship of IL-6 to immune-related cytotoxicity.

## Materials and methods

### Experimental animals

Wistar rats (200–250 g, Charles River Canada) were used for isolation of bone marrow MSCs, and Sprague-Dawley rats (Charles River Canada) were used for the isolation of peripheral blood leucocytes. The study protocol was approved by the Animal Care Committee of the University Health Network and conformed to the ‘Guide for the Care and Use of Laboratory Animals’ published by the US National Institutes of Health (NIH Publication No. 85-23, revised 1985).

### MSC preparation, differentiation and oxidative damage

Mesenchymal stem cells (CD45−/CD34−/CD90.1+/CD29+ by FACS analysis) were isolated from the femurs and tibias of Wistar rats as previously described [2]. After the connective tissue was removed from around the bones, both ends were cut. The bone marrow plugs were flushed with Iscove's Modified Dulbecco's Medium supplemented with 10% foetal bovine serum (FBS), 100 units/ml penicillin G and 0.1 mg/ml streptomycin. Cells were plated and cultured in the same medium. Three days later, the medium was changed and non-adherent cells were discarded. The medium was replaced every 3 days, and the cells were subcultured when confluency exceeded 90%. Mesenchymal stem cells from passages 3 to 5 were used for the studies.

To induce myogenic differentiation, MSCs were cultured with 10 μM 5-azacytidine (5-AZA; Sigma-Aldrich, Oakville, ON, Canada) for 24 hrs and then maintained in normal growth medium for 2 weeks. To induce differentiation towards endothelial or smooth muscle cells, MSCs were cultured for 1 week in media containing 2% FBS with either VEGF (50 ng/ml; R&D Systems, Minneapolis, MN, USA) to induce endothelial characteristics or transforming growth factor-β1 (TGF-β1) (10 ng/ml; R&D Systems) to induce smooth muscle characteristics.

To assess the effects of IL-6 on cell death of differentiated cells without leucocyte co-culture, cellular apoptosis and necrosis were induced in myogenic-differentiated MSCs by treatment with 400 μM of H_2_O_2_ for 48 hrs. Recombinant IL-6 (10 ng/ml; R&D Systems) was added to the cells 1 hr prior to the addition of H_2_O_2_.

### RT-PCR

Total cellular RNA was extracted using Trizol reagent following the manufacturer's instructions. The cDNA was synthesized from 2 μg total RNA using 200 units of SuperScript III reverse transcriptase (Invitrogen) and 1 μl of oligo(dT)_20_ primers (50 μM; Invitrogen, Burlington, ON, Canada). The reverse transcription was performed at 50°C for 60 min. and terminated at 70°C for 15 min. PCR was performed with Taq polymerase for 32 cycles of 95°C for 60 sec., 55°C for 30 sec., and 72°C for 60 sec., with an additional 10 min. at 72°C after completion of the final cycle. The PCR products were fractionated by 1.5% agarose gel electrophoresis. GAPDH was amplified as a reference for quantification of IL-6 mRNA.

### Immunocytochemistry

Immunofluorescent staining was performed to identify cell differentiation markers. In brief, cultured cells were fixed in 2% paraformaldehyde for 10 min., blocked in 5% bovine serum albumin or 5% goat serum, and then incubated with one of the following primary antibodies: myosin heavy chain (MHC; Abcam, Toronto, ON, Canada) for myogenic cells, FLK-1 (Abnova, Taipei, Taiwan) and von Willebrand factor (vWF; Dako, Burlington, ON, Canada) for endothelial cells, and smooth muscle actin (SMA; Sigma-Aldrich), smoothelin (Santa Cruz, Dallas, TX, USA), and smooth muscle myosin heavy chain (SMMHC; Santa Cruz) for smooth muscle cells. Incubation with respective Alexa 488 or 555 conjugated secondary antibodies was carried out at room temperature with light protection for 1 hr. Nuclei were stained with DAPI (Sigma-Aldrich). The cells were examined using a Nikon (Mississauga, ON, Canada) Eclipse TE200 fluorescence microscope.

### LDL staining for endothelial identification

After culturing cells for 7 days, medium was changed and adherent cells were washed with medium and incubated with 10 μg/ml 1,1′-dioctadecyl-3,3,3′,3′-tetramethylindocarbocyanine–labeled acetylated LDL (Di-acLDL; Biomedical Technologies Inc., Stoughton, MA, USA) for 4 hrs. Cells were fixed in 2% paraformaldehyde and counterstained with DAPI.

### Quantification of IL-6

To determine IL-6 levels, cells were cultured and differentiated in six-well plates as mentioned above. After 48 hrs, the culture medium was removed and centrifuged at 80 *g* for 5 min. The amount of IL-6 in the culture medium was measured by ELISA (R&D Systems) according to the manufacturer's instructions and expressed as pg/mg total protein.

### Flow cytometry

Annexin V-FITC and propidium iodide (PI; BD Biosciences, Mississauga, ON, Canada) staining was used to evaluate cell apoptosis and necrosis following the manufacturer's instructions. For the leucocyte co-culture studies, culture dishes were carefully washed multiple times with PBS to remove the leucocytes prior to staining. In brief, 5 μl annexin V-FITC and/or 5 μl PI was added to 1 × 10^5^ cells in 100 μl binding buffer. The mixture was gently vortexed and incubated for 15 min. at room temperature in the dark, and 400 μl of binding buffer was added to each sample. The samples were analyzed within 1 hr by flow cytometry. Quantification of cell apoptosis (annexin V positive) and cell necrosis (both PI positive and PI and annexin V dual-positive cells) was performed using an FC500 flow cytometer (Beckman Coulter, Mississauga, ON, Canada).

### Leucocyte-mediated cytotoxicity

Mixed peripheral blood leucocytes were isolated from the blood of Sprague-Dawley rats using gradient centrifugation (Sigma-Aldrich) according to the manufacturer's protocol. Peripheral blood leucocytes (3 × 10^6^) were co-cultured with differentiated or undifferentiated allogeneic MSCs (3 × 10^5^) from Wister rats in six-well plates in the presence or absence of 10 ng/ml recombinant IL-6 (R&D Systems). After 2 days, leucocyte-mediated cytotoxicity of the MSCs was assessed by collecting the supernatant and measuring the lactate dehydrogenase (LDH) released from the damaged cells using a cytotoxicity detection kit (Roche Applied Science, Laval, QC, Canada). Lactate dehydrogenase activity is directly proportional to the optical density measured at 490 nm with a reference filter of 620 nm.

### Statistical analyses

Data are expressed as mean ± SD and were compared between groups using unpaired *t*-tests or one-way anova followed by a Tukey post-test (if the F ratio was statistically significant). Differences were considered significant at *P* < 0.05.

## Results

### Myogenic differentiation of MSCs decreased cellular IL-6

**Fig. 1:**
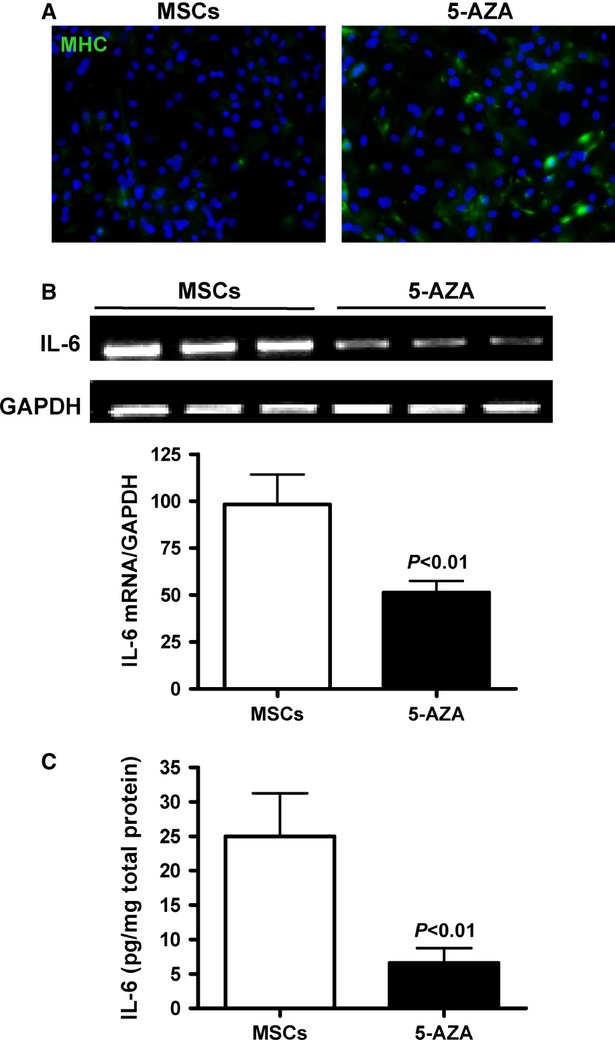
Downregulation of IL-6 by myogenic differentiation of mesenchymal stem cells (MSCs). Bone marrow MSCs were treated with 5-AZA for 24 hrs to induce differentiation to myogenic cells. (**A**) Immunostaining showed MHC protein expression in the 5-AZA–treated cells (200 ×). (**B**) RT-PCR showed that IL-6 mRNA expression was significantly reduced in myogenic-differentiated cells compared to undifferentiated MSCs (*n* = 6/group). (**C**) IL-6 protein levels were significantly lower in myogenic-differentiated cells compared to undifferentiated MSCs as measured by ELISA (*n* = 5/group).

To examine the changes in IL-6 related to cell differentiation, rat MSCs were treated with 5-AZA for 24 hrs and cultured for 2 weeks to induce myogenic differentiation. Immunostaining showed the expression of MHC protein in the myogenic-differentiated cells (Fig. 1A). IL-6 in undifferentiated MSCs and 5-AZA–treated cells was analyzed by RT-PCR and ELISA. The IL-6 mRNA expression decreased 47.7% (Fig. 1B) and IL-6 protein decreased 73.4% with myogenic differentiation (Fig. 1C).

### IL-6 downregulation was differentiation dependent but not cell phenotype dependent

**Fig. 2:**
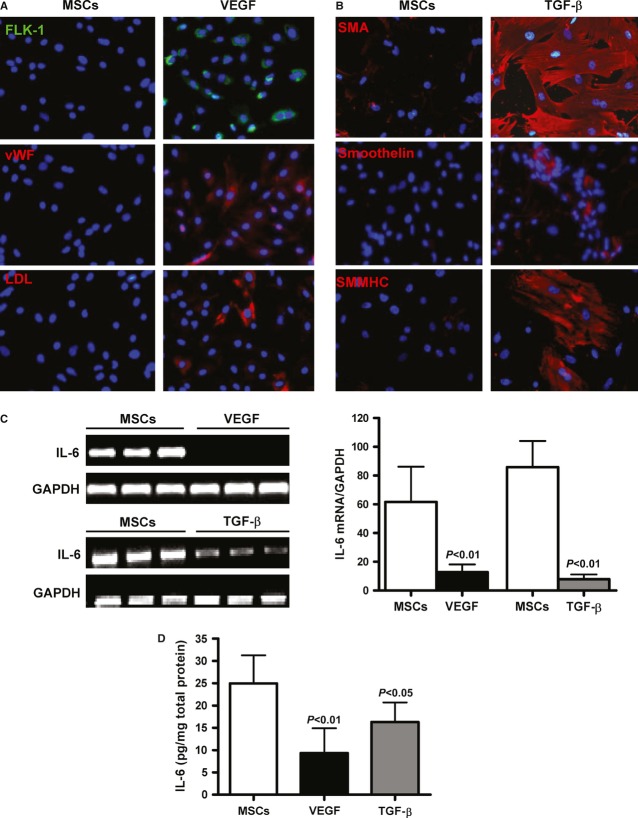
Downregulation of IL-6 was differentiation dependent but not cell phenotype dependent. Mesenchymal stem cells (MSCs) were treated with VEGF or TGF-β for 7 days to induce differentiation to endothelial or smooth muscle cells, respectively. (**A**) Endothelial cell differentiation was confirmed by immunostaining for FLK-1 and vWF as well as by the uptake of Di-acLDL (200 ×). (**B**) Smooth muscle cell differentiation was confirmed by immunostaining for SMA, smoothelin, and SMMHC (200 ×). (**C**) RT-PCR analysis confirmed the downregulation of IL-6 mRNA expression by VEGF-induced endothelial differentiation and TGF-β–induced smooth muscle cell differentiation (*n* = 6/group). (**D**) IL-6 protein levels were significantly lower in endothelial-differentiated and smooth muscle–differentiated cells compared to undifferentiated MSCs as measured by ELISA (*n* = 4–5/group).

To investigate whether downregulation of cellular IL-6 in relation to MSC differentiation was phenotype dependent, MSCs were also induced to differentiate to endothelial cells or smooth muscle cells by treatment with VEGF or TGF-β, respectively. Endothelial cell differentiation was confirmed by immunostaining for FLK-1 and vWF as well as by the uptake of Di-acLDL (Fig. 2A). Smooth muscle cell differentiation was confirmed by immunostaining for SMA, smoothelin and SMMHC (Fig. 2B). RT-PCR analysis showed a significant decrease in IL-6 mRNA expression in VEGF-induced endothelial cells compared with undifferentiated MSCs (*P* < 0.01, Fig. 2C), and ELISA showed that IL-6 protein levels also significantly decreased in the differentiated cells (*P* < 0.01, Fig. 2D). An almost identical pattern was observed in the MSCs differentiated to smooth muscle cells by treatment with TGF-β (Fig. 2C and D). These results indicate that MSC differentiation to all three cell phenotypes resulted in downregulation of IL-6.

### Downregulation of IL-6 in myogenic-differentiated cells was associated with increased leucocyte-mediated cytotoxicity

**Fig. 3:**
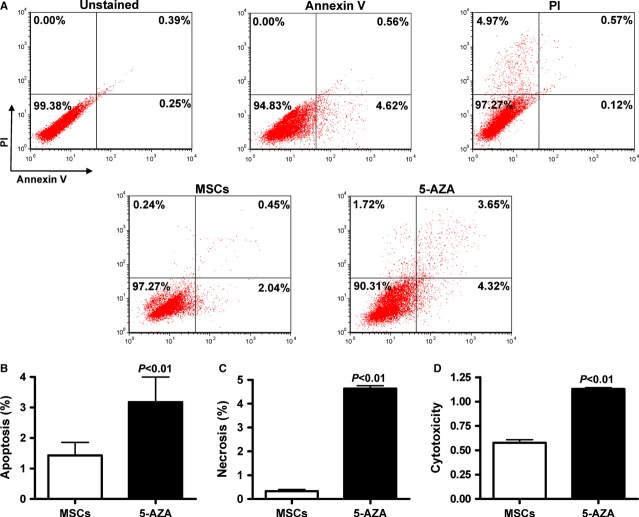
Downregulation of IL-6 in myogenic-differentiated cells was associated with increased cell death and leucocyte-mediated cytotoxicity. Peripheral leucocytes from Sprague-Dawley rats were co-cultured for 2 days with either undifferentiated mesenchymal stem cells (MSCs) or myogenic differentiated (5-AZA–treated) MSCs from Wistar rats. (**A**) Representative dual-colored annexin V (apoptotic cells) and PI (necrotic cells) flow cytometry plots of undifferentiated and 5-AZA–treated MSCs. (**B**) The percentage of apoptotic cells (annexin V positive) was significantly greater in myogenic-differentiated cells than control MSCs (*n* = 6/group). (**C**) The percentage of necrotic cells (PI positive and PI/annexin V dual positive) was significantly greater in myogenic-differentiated cells than control MSCs (*n* = 5–6/group). (**D**) Greater leucocyte-mediated cytotoxicity (LDH release) was observed in the co-cultures containing myogenic-differentiated cells compared with undifferentiated MSCs (*n* = 5–6/group).

To evaluate the effects of decreased IL-6 on MSC immunoprivilege, a co-culture system was established to evaluate allogeneic leucocyte-mediated cytotoxicity. Either undifferentiated MSCs or myogenic-differentiated MSCs from Wistar rats were co-cultured for 2 days with peripheral leucocytes isolated from Sprague-Dawley rats. Cellular apoptosis and necrosis were evaluated using flow cytometry after annexin V and PI staining (Fig. 3A). The percentage of apoptotic cells (annexin V positive) was significantly higher in the myogenic cells than in the undifferentiated MSCs (*P* < 0.01, Fig. 3B). The percentage of necrotic cells (both PI positive and PI/annexin V double positive) was also significantly greater in the myogenic cells compared with the undifferentiated MSCs (*P* < 0.01, Fig. 3C). Cytotoxicity was evaluated by LDH release into the culture medium. Higher amounts of LDH were found in the culture media of myogenic-differentiated cells compared with undifferentiated MSCs (*P* < 0.01, Fig. 3D). These results suggest that the myogenic cells lost their immune privilege and underwent leucocyte-mediated cytotoxicity after differentiation and downregulation of IL-6.

### Leucocyte-mediated cytotoxicity was differentiation dependent but not cell phenotype dependent

**Fig. 4:**
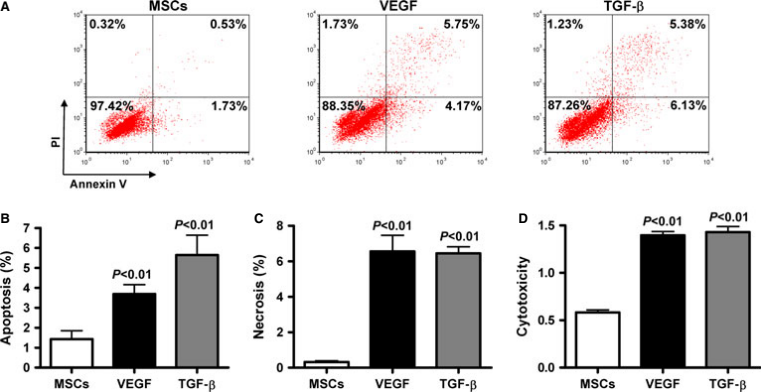
Leucocyte-mediated cytotoxicity was differentiation dependent but not cell phenotype dependent. Mesenchymal stem cells (MSCs) were treated with VEGF or TGF-β to induce differentiation to endothelial or smooth muscle cells, respectively. Allogeneic peripheral leucocytes were co-cultured for 2 days with either undifferentiated or differentiated MSCs. (**A**) Representative dual-colored annexin V and PI flow cytometry plots of undifferentiated MSCs and VEGF- or TGF-β–treated MSCs. (**B** and **C**) Both cell apoptosis (annexin V positive, B, *n* = 6/group) and cell necrosis (PI positive and PI/annexin V dual positive, C, *n* = 5–6/group) were significantly greater in the differentiated cells than in the control MSCs. (**D**) Leucocyte-mediated cytotoxicity (LDH release) was greater in the co-cultures containing endothelial-differentiated and smooth muscle–differentiated cells compared to control MSCs (*n* = 5–6/group).

To investigate whether different cell phenotypes had divergent effects on leucocyte-mediated cytotoxicity, we employed the same co-culture system to examine cell death for MSCs differentiated to endothelial cells and smooth muscle cells. As stated earlier, cell apoptosis and necrosis were evaluated by annexin V and PI flow cytometry (Fig. 4A). The percentage of apoptotic cells in MSC-derived endothelial and smooth muscle cells was significantly greater compared with that in the undifferentiated MSCs (*P* < 0.01, Fig. 4B). Similarly, cell necrosis was significantly greater after both VEGF and TGF-β treatment (*P* < 0.01, Fig. 4C). Cytotoxicity evaluation by LDH release revealed that both VEGF-induced endothelial cells and TGF-β–induced smooth muscle cells exhibited significantly greater cell damage compared with untreated MSCs (*P* < 0.01, Fig. 4D). These results confirm that leucocyte-mediated cytotoxicity was dependent on differentiation but not on cell phenotype.

### Restoration of IL-6 partially rescued differentiated MSCs from cell death

**Fig. 5:**
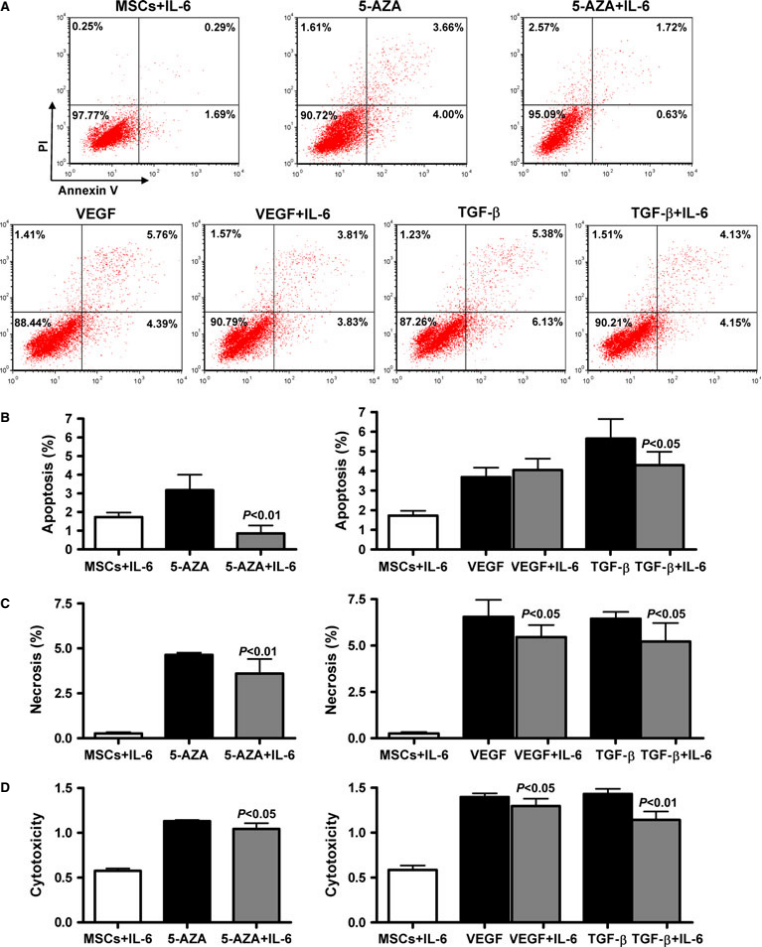
Restoration of IL-6 partially rescued the differentiated cells from cell death. Mesenchymal stem cells (MSCs) were treated with 5-AZA, VEGF, or TGF-β to induce differentiation to myogenic, endothelial, or smooth muscle cells, respectively. Allogeneic peripheral leucocytes were co-cultured for 2 days with either undifferentiated or differentiated MSCs in the presence or absence of IL-6. (**A**) Representative dual-colored flow cytometry plots of undifferentiated MSCs plus IL-6 and MSCs treated with 5-AZA, VEGF, or TGF-β, with or without IL-6. (**B**) Quantification of cell apoptosis (annexin V positive) showed that IL-6 significantly reduced cell apoptosis of myogenic-differentiated cells and smooth muscle–differentiated cells but did not have an effect on endothelial-differentiated cells (*n* = 6/group). (**C**) Cell necrosis (PI positive and PI/annexin V dual positive) was significantly reduced in all three differentiated cell types treated with IL-6 (*n* = 5–6/group). (**D**) Cytotoxicity (LDH release) in all three differentiated cell types was significantly reduced in the co-cultures containing IL-6 (*n* = 5–6/group).

To confirm that IL-6 plays an important role in differentiation-associated cell death in this co-culture system, MSC-derived myogenic cells, endothelial cells or smooth muscle cells were co-cultured with allogeneic leucocytes to examine the leucocyte-mediated cytotoxicity with and without the addition of IL-6. As stated earlier, cell apoptosis and necrosis were evaluated by annexin V and PI flow cytometry (Fig. 5A). We found that the addition of recombinant IL-6 to the myogenic-differentiated cells decreased apoptosis to a level similar to that of undifferentiated MSCs (*P* < 0.01, Fig. 5A). Cell necrosis was also significantly lower in myogenic-differentiated cells treated with IL-6 (*P* < 0.01, Fig. 5B). This trend was also observed in the MSC-derived endothelial and smooth muscle cells. The addition of IL-6 rescued the differentiated cells from undergoing cell apoptosis and cell death (*P* < 0.05, Fig. 5B and C), with the exception of the VEGF-induced endothelial cells, which did not show a decrease in apoptosis. The leucocyte-mediated cytotoxicity results paralleled these observations. The restoration of IL-6 partially rescued the differentiated cells from cell damage compared with differentiated cells without the addition of IL-6, regardless of cell phenotype (*P* < 0.05, Fig. 5D).

**Fig. 6:**
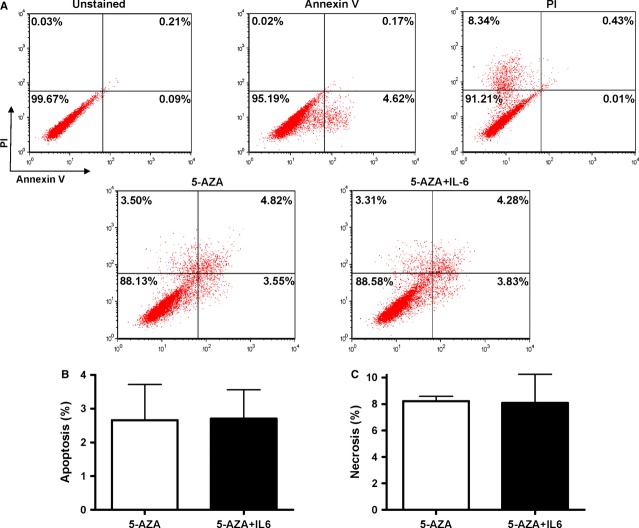
IL-6 did not rescue myogenic-differentiated cells from H_2_O_2_-induced cell death. Mesenchymal stem cells (MSCs) were treated with 5-AZA to induce differentiation to myogenic cells. Cellular apoptosis and necrosis were induced by H_2_O_2_ treatment. IL-6 was added to the cells 1 hr prior to H_2_O_2_. (**A**) Representative dual-colored flow cytometry plots of MSCs treated with 5-AZA, with and without IL-6. (**B**) Quantification of cell apoptosis (annexin V positive) showed that IL-6 had no effect on apoptosis of myogenic-differentiated cells. (**C**) Quantification of cell necrosis (PI positive and PI/annexin V dual positive) demonstrated that IL-6 had no effect (*n* = 6–7/group).

To ensure that the observed decrease in differentiated cell death following treatment with IL-6 was related to the decrease in leucocyte-mediated cytotoxicity and not merely the result of IL-6 suppression of apoptosis and necrosis, we assessed cell death in myogenic-differentiated cells treated with H_2_O_2_ rather than co-cultured with leucocytes (Fig. 6). We found that the addition of recombinant IL-6 to the H_2_O_2_-treated myogenic-differentiated cells did not affect apoptosis (Fig. 6B) or necrosis (Fig. 6C).

These data confirm the significant role of IL-6 in leucocyte-mediated cytotoxicity and differentiation-related cell death.

## Discussion

In this study, we showed for the first time that IL-6 was significantly downregulated in MSCs following myogenic, endothelial and smooth muscle cell differentiation. We found that both IL-6 downregulation and leucocyte-mediated cytotoxicity were differentiation dependent but not cell phenotype dependent. Furthermore, the restoration of IL-6 levels at least partially rescued the differentiated cells from leucocyte-mediated cell injury and death.

Bone marrow MSCs are multipotent and can restore cardiac function following implantation into damaged myocardial tissue [10, 12, 21]. Unfortunately, the patients who need cell therapy the most are older, and their stem cells are dysfunctional [8, 9]. Allogeneic MSCs derived from young healthy donors offer the promise of cardiac restoration for aged individuals [11]. Allogeneic MSCs provide an ‘off-the-shelf’ product because of their ease of preparation [21, 22]. Therefore, MSCs are an ideal candidate for cell therapy to restore cardiac function in patients who have suffered an extensive MI. Allogeneic MSC transplantation is currently undergoing clinical trials, but the long-term benefits of this approach have not yet been established, and the long-term fate of the cells remains questionable. We previously reported that allogeneic MSC transplantation improved cardiac function early after implantation into the infarcted rat heart, but following myogenic differentiation, the cells were no longer immunoprivileged and were rejected [2]. Cellular rejection was followed by the deterioration of ventricular function late after cell injection [2]. The current study was initiated to explore the mechanisms associated with this differentiation-induced loss of immune privilege. New strategies that permit allogeneic MSCs to maintain their immune privilege despite differentiation may provide a new alternative to secure their lasting beneficial effects on cardiac function after MI. Because allogeneic MSCs offer the best hope to regenerate the aged heart after injury, this treatment may have substantial clinical effects.

The immunoprivilege of MSCs is maintained by their unique MHC expression pattern and their secretion of soluble immunosuppressive factors, including IDO and PGE2 [14, 16]. We found that *in vitro* differentiation of allogeneic MSCs was accompanied by changes in the expression of the MHC cell surface antigens related to immune rejection [2], and in a more recent study, we found that preserving the PGE2 level prevented rejection of implanted allogeneic MSCs and restored post-infarction ventricular function (Sanjiv Dhingra, Peng Li, Xi-Ping Huang, Jian Guo, Jun Wu, Anton Mihic, Shu-Hong Li, Wang-Fu Zang, Daniel Shen, Richard D. Weisel, Pawan K. Singal, Ren-Ke Li, submitted). Another MSC-secreted factor, IL-6, which has been regarded as a pro-inflammatory cytokine, has been reported to suppress T cell proliferation and attenuate local inflammation [17, 18]. IL-6--type cytokines derived from MSCs have recently been reported to modulate the immune system through JAK-STAT3 signaling [23]. Based on these reports, IL-6 appears to be involved in a series of immunosuppressive activities. As MSCs constitutively secrete IL-6 [15], we postulated that its reduction may be one trigger responsible for the rejection of allogeneic MSCs after implantation. We investigated the expression pattern of IL-6 after MSC differentiation and the influence of IL-6 on differentiated cell survival. We found that IL-6 levels decreased significantly following the differentiation of MSCs to myogenic, endothelial and smooth muscle cell types. The mixed-leucocyte co-culture studies revealed that cell apoptosis and necrosis were significantly increased after MSC differentiation and allogeneic leucocytes were activated by the differentiated cells, resulting in increased cytotoxicity. These *in vitro* data suggest that downregulation of IL-6 in MSCs following differentiation may play a role in eliciting leucocyte-mediated cell damage. Consistent with these findings, we previously demonstrated that late after MSC implantation into the infarcted heart, the cells lost their immune privilege following differentiation, became susceptible to leucocyte cytotoxicity, and were eventually rejected [2]. The downregulation of IL-6 associated with the differentiation of MSCs could be another mechanism responsible for allogeneic cell rejection after differentiation.

Therefore, we proposed that administration of recombinant IL-6 may protect the differentiated cells from the cytotoxicity of activated allogeneic leucocytes in our mixed-leucocyte co-culture system. We found that cell apoptosis and necrosis after myogenic and smooth muscle cell differentiation were significantly reduced in the presence of IL-6. Correspondingly, cytotoxicity was also significantly decreased. Similar effects of IL-6 restoration were observed on endothelial-differentiated cells, but the degree of apoptosis was not reduced with IL-6. We speculate that IL-6 may provide different levels of protection for different cell types.

Taken together, our results reveal for the first time that IL-6 plays an important role in preventing allogeneic leucocyte-mediated cell damage after differentiation of MSCs. Our findings suggest that reduced IL-6 levels contribute to the rejection of allogeneic MSCs after implantation. Restoration of IL-6 in differentiated MSCs could enhance their long-term capacity for tissue repair and regeneration.
